# Tolerability of Opioid Analgesia for Chronic Pain: A Network Meta-Analysis

**DOI:** 10.1038/s41598-017-02209-x

**Published:** 2017-05-17

**Authors:** Zengdong Meng, Jing Yu, Michael Acuff, Chong Luo, Sanrong Wang, Lehua Yu, Rongzhong Huang

**Affiliations:** 1 Department of Orthopaedics, First People’s Hospital of YunNan Province, YunNan, P.R. China; 2 Department of Rehabilitation Medicine, The second Affiliated Hospital of Chongqing Medical University, Chongqing, P.R. China; 3 Department of Pain Medicine, The second Affiliated Hospital of Chongqing Medical University, Chongqing, P.R. China; 4 Department of Preventive Medicine, Keck School of Medicine, University of Southern California, Los Angeles, CA USA; 5 Rusk Rehabilitation Center, University of Missouri School of Medicine, Columbia, Missouri USA

## Abstract

Aim of this study was to study the tolerability of opioid analgesia by performing a network meta-analysis (NMA) of randomized-controlled trials (RCTs) which investigated effectiveness of opioids for the management of chronic pain. Research articles reporting outcomes of RCT/s comparing 2 or more opioid analgesics for the management of chronic pain were obtained by database search. Bayesian NMAs were performed to combine direct comparisons between treatments with that of indirect simulated evidence. Study endpoints were: incidence of adverse events, incidence of constipation, trial withdrawal rate, and patient satisfaction with treatment. Outcomes were also compared with conventional meta-analyses. Thirty-two studies investigating 10 opioid drugs fulfilled the eligibility criteria. Tapentadol treatment was top-ranking owing to lower incidence of overall adverse events, constipation, and least trial withdrawal rate. Tapentadol was followed by oxycodone-naloxone combination in providing better tolerability and less trial withdrawal rate. Patient satisfaction was found to be higher with oxycodone-naloxone followed by fentanyl and tapentadol. These results were in agreement with those achieved with conventional meta-analyses. Tapentadol and oxycodone-naloxone are found to exhibit better tolerability characteristics in comparison with other opioid drugs for the management of chronic pain and are associated with low trial withdrawal rate and better patient satisfaction.

## Introduction

Chronic pain is defined as the pain lasting for more than 3 months and is characterized by a continuum with an inherently uncertain prognosis^1, 2^. Pain in its severe form significantly affects physical and mental health^3, 4^. Up to 70% of cancer patients suffer from etiological or iatrogenic pain^5^. In US alone, approximately 100 million individuals suffer from chronic pain^6^. At least 20% of adult population in western countries experience chronic pain at any stage of life^7–9^ and approximately 40% exhibit their dissatisfaction over adequate pain management^3^.

Opioid receptor agonists are widely used to treat chronic pain and are considered as the standard of care^10, 11^. Short-term use of opioids for chronic pain is associated with reduction in pain intensity, improved functional outcomes but with significant side effects and discontinuation rates^12–15^. Recently issued Center for Disease Control and Prevention (CDC) guidelines for prescribing opioids for chronic pain stress for risk assessment as a continuous process during opioid therapy^16, 17^.

Whereas, use of opioids receptor agonists (hereinafter opioids) for chronic cancer and non-cancer pain is reviewed^18, 19^, a head-to-head comparison of the risks in terms of tolerability and adherence associated with different types of opioid analgesics is not available in literature. We have carried out a systematic review of literature in order to identify RCTs which addressed this issue and performed network meta-analyses to evaluate the tolerability characteristics, trial withdrawal rate and patient satisfaction with opioid treatment for chronic pain and have compared the outcomes with conventional meta-analyses.

## Method

This systematic review and meta-analysis was performed by following the Cochrane Handbook for Systematic Reviews guidelines^20^ and is reported in accordance with the Preferred Reporting Items for Systematic reviews and Meta-Analysis (PRSIMA) statement^21^.

### Literature Search

Embase, Ovid SP, and PubMed/Medline electronic databases were searched for the relevant RCT reports published during the inception date of respective database and June 2016. Primary MeSH/keywords used were: Opioid-chronic pain, opioid agonist-chronic pain, opioid receptor agonist-chronic pain, buprenorphine-chronic pain, fentanyl-chronic pain, hydromorphone-chronic pain, methadone-chronic pain, morphine-chronic pain, naloxone-chronic pain, oxycodone-chronic pain, oxymorphone-chronic pain, tapentadol-chronic pain, tramadol-chronic pain. Secondary MeSH/keywords used with any of primary terms were: osteoarthritis, low back pain, cancer pain, musculoskeletal pain, neuropathic pain, and nociceptive. Tertiary MeSH/keywords used with any of primary and/or secondary combination/s were: efficacy, safety, tolerability, randomized trial, trial withdrawal and patient outcomes. Reference lists of the selected articles were also seen for additional study reports. Eligibility of the trials was assessed by two researchers independently who then unified the list of selected studies mutually. Inter-rater reliability was good (kappa = 0.94).

### Eligibility Criteria

To be included in the meta-analysis a study, (a) had to be a RCT recruiting patients with chronic pain (cancer or non-cancer) to treat with an opioid drug either alone or in combination with N-methyl D-aspartic acid (NMDA) receptor antagonist for the management of chronic pain; (b) evaluated the outcomes against a comparator opioid either alone or in combination with NMDA receptor antagonist treated group; and (c) reported at least one of the following endpoints: (1) incidence of adverse events; (2) incidence of constipation; (3) trial withdrawal rate; and (4) patient satisfaction (satisfaction, convenience, improvement, or relief; patient’s excellent or good response to an appraisal regarding the effectiveness of opioid therapy using a Likert scale or a dichotomous tool). A study/arm was excluded if tested an opioid or combination of opioids against a placebo group; used an opioid in combination with a non-opioid analgesic drug other than NMDA) receptor antagonist; intervened for cancer breakthrough pain or non-cancer acute pain; or had single-arm design.

### Data and Analyses

Data were extracted by two authors independently who then unified the output by attaining consensus on dubious situations. Incidence of adverse events was prespecified as the primary outcome measure, whereas the incidence of constipation, trial withdrawal rate and patient satisfaction with treatment were used as secondary endpoints. For the assessment of the quality of included studies, Cochrane Collaboration’s Tool for the Assessment of Quality of RCTs was used.

Network meta-analyses utilized Bayesian random effects Poisson regression based probabilistic modelling with Markov-Chain Monte-Carlo method. This model preserves randomized treatment comparisons within the trials. Model used total number of events and accumulated patients/patient-weeks to estimate odds ratios. Prior distribution for treatment effects was none-informative. Model involved calculations on 50,000 iterations with 20,000 iterations as burn-in. Statistical heterogeneity between the trials was estimated from between-trial variance (τ^2^) of the posterior distribution. Ranking probabilities were examined either with rankograms or by obtaining surface under the cumulative ranking (SUCRA; a simple numerical summary of the probabilities ranging between 100% (the best) and 0% (the worst). Statistical procedures were performed with WinBUGS software (version 1.4) by using NetMetaXL interface^22^.

Convergence of the iterative simulations was assessed by using the Brooks-Gelman-Rubin method by comparing within-chain and between-chain variances to calculate the potential scale reduction factor (PSRF; good convergence was considered when PSRF was close to 1) as well as by noting whether the Monte Carlo error is less than 5% of the standard deviation of the effect estimates and between-study variance. Assessment of inconsistency of direct and indirect evidence of the treatment network was performed by comparing the deviance residuals and deviance information criterion (DIC) statistics in fitted consistency and inconsistency models^23^. For the visual examination, the posterior mean deviance of the individual data points in the inconsistency model were plotted against their posterior mean deviance in the consistency model to identify possible loops in the network representing inconsistency.

Conventional meta-analyses were carried out with Stata software (Stata Corporation, Texas, USA) by using the numeric values of the study endpoints to achieve individual and overall odds ratios between the comparator groups. A particular opioid drug was compared with all possible comparator opioids. Between study heterogeneity was assessed by the chi-squared (chi^2^) and I-squared (I^2^) indices. A p < 0.1 and I^2^ > 50% were considered to indicate significant statistical heterogeneity.

## Results

**Figure 1:**
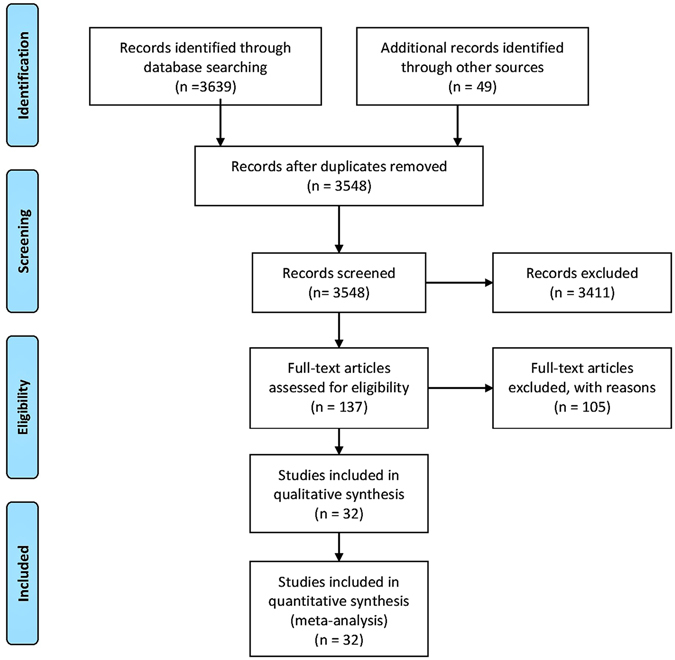
A flowchart of the study screening and selection process.

During the literature search over 3500 titles and abstracts screened of which 137 were selected for full-text research article retrieval. Observation of the eligibility criteria led to the final selection of 32 research articles^24–55^ reporting the outcomes of the RCTs (Fig. 1).

Important characteristics of the included trials are presented in Appendix S2. In general, trials were of moderate to high methodological quality. Appropriate methods of allocation concealment were described for 18 trials (56%). Blinding of outcome assessment was also reported for 18 trial (56%). An assessment summary is presented in Table S1.

Overall network consisted of 10 opioid treatment regimens. These were: buprenorphine, fentanyl, hydromorphone, methadone, morphine, oxycodone, oxycodone-naloxone, oxymorphone, tapentadol, and tramadol. Visual illustrations showing the network of treatment regimens for study endpoints are presented as Fig. (S1a–c). In all analyses, convergence of the iterative simulations was reached.

No significant inconsistency between direct and indirect evidence was observed for any of the study endpoints. Figure S2a–d show the plotting of posterior mean deviance of the individual data points in the inconsistency model against their posterior mean deviance in the consistency model for all four endpoints.

### Incidence of Adverse Events

Conventional meta-analysis revealed that tapentadol treatment was associated with significantly (p < 0.00001) less incidence of adverse events (AEs) in comparison with other opioids. There was no significant difference in the incidence of AEs between oxycodone-naloxone, morphine, fentanyl, oxycodone, or tramadol and their comparator opioids (Table 1 and Figure S3a).

**Table 1:** Outcomes of the conventional meta-analyses showing the odds ratios between a selected opioids and other opioids in study endpoints.

Opioid vs others	OR [95% CI]	p	I^2^	Opioid vs others	OR [95% CI]	p	I^2^
**Incidence of adverse events**				**Incidence of constipation**			
TAP	0.81 [0.73, 0.90]	<0.00001	36%	TAP	0.56 [0.44, 0.70]	<0.00001	54%
OXY	0.89 [0.78, 1.02]	=0.087	0%	MOR	0.57 [0.38, 0.86]	=0.007	0%
TRA	0.87 [0.65, 1.17]	=0.352	71%	OXN	0.68 [0.46, 0.99]	=0.042	64%
OXN	0.92 [0.78, 1.10]	=0.369	50%	FENT	0.79 [0.65, 0.96]	=0.015	0%
FENT	0.96 [0.88, 1.04]	=0.278	0%	TRA	0.75 [0.31, 1.79]	=0.515	37%
MOR	0.99 [0.88, 1.11]	=0.842	0%	OXY	0.94 [0.82, 1.09]	=0.413	0%
**Trial withdrawal rate**				**Patient satisfaction**			
TAP	0.64 [0.47, 0.88]	=0.006	84%	OXN	1.70 [1.46, 1.98]	<0.00001	0%
OXN	0.86 [0.74, 1.00]	=0.057	0%	FENT	1.31 [0.76, 2.24]	=0.332	0%
FENT	0.80 [0.51, 1.27]	=0.350	53%	TAP	1.10 [0.96, 1.25]	=0.183	0%
OXY	0.87 [0.72, 1.04]	=0.123	0%	MOR	1.06 [0.87, 1.30]	=0.543	0%
HYD	0.95 [0.77, 1.17]	=0.645	0%	HYD	1.05 [0.83, 1.33]	=0.694	0%
BUP	0.97 [0.66, 1.43]	=0.877	0%	BUP	1.02 [0.81, 1.29]	=0.855	0%

Abbreviations: BUP, buprenorphine; CI, confidence interval; FENT, fentanyl; HYD, hydromorphone; MOR, morphine; OR, odds ration; OXN, oxycodone-naloxone; OXY, oxycodone; TAP, tapentadol; TRA, tramadol.

In NMA of the incidence of AEs, total number of interventions were 9 which were carried out in 25 studies involving 280,292 patient-weeks. Network, intervention, and direct comparison characteristics of this NMA are given in Tables S2–4. Oxycodone was the most investigated opioid (17 studies; compared with 9 different opioids) followed by morphine and hydromorphone (7 studies each). This NMA revealed that least incidence of AEs was associated with tapentadol treatment followed by oxycodone-naloxone, tramadol and oxycodone (Table 2). Probability for ranking top among the opioids was 0.698 for tapentadol and the probability for ranking second was 0.26 for oxycodone-naloxone combination (Figure S4a). The SUCRA probabilities for various opioids were: tapentadol (0.945), oxycodone-naloxone (0.652), tramadol (0.651), oxycodone (0.507), fentanyl (0.467), morphine (0.437), hydromorphone (0.420), buprenorphine (0.368), and oxymorphone (0.054) (Figure S5a).

**Table 2:** League table showing the odds of the incidence of **ADVERSE EVENTS** with treatment of various opioids.

**Tapentadol**								
0.84 (0.68–1.05)	**Oxycodone-Naloxone**							
0.85 (0.54–1.36)	1.01 (0.64–1.63)	**Tramadol**						
0.79 (0.69–0.92)	0.94 (0.79–1.13)	0.93 (0.60–1.43)	**Oxycodone**					
0.77 (0.55–1.06)	0.92 (0.64–1.27)	0.91 (0.52–1.50)	0.98 (0.71–1.30)	**Fentanyl**				
0.77 (0.59–1.00)	0.92 (0.70–1.19)	0.90 (0.55–1.45)	0.97 (0.78–1.21)	0.99 (0.80–1.28)	**Morphine**			
0.76 (0.61–0.97)	0.91 (0.71–1.17)	0.90 (0.56–1.43)	0.97 (0.80–1.16)	0.99 (0.74–1.37)	0.99 (0.80–1.24)	**Hydromorphone**		
0.74 (0.51–1.10)	0.88 (0.60–1.32)	0.87 (0.67–1.12)	0.94 (0.66–1.34)	0.96 (0.62–1.56)	0.96 (0.64–1.47)	0.97 (0.66–1.45)	**Buprenorphine**	
0.55 (0.37–0.83)	0.66 (0.44–0.99)	0.65 (0.36–1.14)	0.70 (0.48–1.01)	0.71 (0.45–1.18)	0.72 (0.47–1.11)	0.72 (0.48–1.09)	0.74 (0.44–1.24)	**Oxymorphone**

OR <1 means the treatment in top left is better.

### Incidence of constipation

Conventional meta-analysis revealed that tapentadol (p < 0.00001), morphine (p = 0.007), fentanyl (p = 0.015), and oxycodone-naloxone (p = 0.042) treatment was associated with significantly less incidence of constipation in comparison with their comparator opioids. There was no significant difference in the incidence between oxycodone or tramadol and their comparator opioids (Table 1 and Figure S3b).

In the NMA, total number of interventions were 9 which were carried out in 25 studies in which 2831 events occurred in 286,864 patient-weeks. Network, intervention, and direct comparison characteristics of this NMA are given in Tables S2–4. Oxycodone was the most investigated opioid (17 studies; compared with 7 different opioids) followed by oxycodone-naloxone (7 studies) and tapentadol (6 studies). This NMA revealed that least incidence of constipation was associated with tapentadol treatment followed by oxycodone-naloxone, fentanyl and tramadol (Table 3). Probability for ranking top among the opioids was 0.516 for tapentadol whereas the probability for ranking second best was 0.4464 for oxycodone-naloxone combination (Figure S4b). SUCRA probabilities for various opioids were: tapentadol (0.913), oxycodone-naloxone (0.829), fentanyl (0.641), tramadol (0.598), morphine (0.389), oxycodone (0.373), buprenorphine (0.364), oxymorphone (0.268) and hydromorphone (0.126) (Figure S5b).

**Table 3:** League table showing the odds of causing **CONSTIPATION** by the various opioids.

**Tapentadol**								
0.90 (0.60–1.26)	**Oxycodone-Naloxone**							
0.69 (0.37–1.24)	0.76 (0.44–1.41)	**Fentanyl**						
0.68 (0.21–2.23)	0.75 (0.23–2.57)	0.99 (0.27–3.60)	**Tramadol**					
0.52 (0.33–0.76)	0.58 (0.40–0.85)	0.76 (0.47–1.16)	0.77 (0.23–2.53)	**Morphine**				
0.51 (0.38–0.64)	0.57 (0.43–0.76)	0.75 (0.42–1.26)	0.75 (0.23–2.37)	0.98 (0.71–1.35)	**Oxycodone**			
0.50 (0.19–1.26)	0.56 (0.22–1.45)	0.74 (0.25–2.07)	0.74 (0.34–1.53)	0.97 (0.37–2.50)	0.99 (0.40–2.42)	**Buprenorphine**		
0.45 (0.22–0.87)	0.50 (0.25–1.03)	0.65 (0.27–1.50)	0.65 (0.17–2.47)	0.86 (0.42–1.78)	0.88 (0.46–1.68)	0.88 (0.29–2.70)	**Oxymorphone**	
0.40 (0.25–0.59)	0.45 (0.29–0.66)	0.59 (0.31–0.99)	0.59 (0.17–1.92)	0.77 (0.52–1.08)	0.78 (0.55–1.07)	0.79 (0.30–2.11)	0.89 (0.42–1.82)	**Hydromorphone**

OR <1 means the treatment in top left is better.

### Trail withdrawal rate

Conventional meta-analysis revealed that tapentadol (p = 0.006) and oxycodone-naloxone (p = 0.057) treatment had significantly less odds of trial withdrawal rate in comparison with other opioids. There was no significant difference in the odds ratios between oxycodone, fentanyl, hydromorphone, buprenorphine and their comparator opioids (Table 1 and Figure S3c).

In NMA, total number of interventions were 10 which were carried out in 27 studies in 12,207 patients of which 4822 withdrew. Network, intervention, and direct comparison characteristics of this NMA are given in Tables S2–4. Oxycodone was the most investigated opioid (18 studies; compared with 7 different opioids) followed by morphine (8 studies), oxycodone-naloxone and tapentadol (7 studies each). This NMA revealed that least trial withdrawal was associated with tapentadol treatment followed by oxycodone-naloxone, fentanyl and hydromorphone (Table 4). Probability for ranking top among the opioids was 0.76 for tapentadol whereas the probability for ranking second best was 0.224 for oxycodone-naloxone combination (Figure S4c). SUCRA probabilities for various opioids were: tapentadol (0.961), oxycodone-naloxone (0.669), fentanyl (0.661), hydromorphone (0.543), oxycodone (0.536), buprenorphine (0.406), methadone (0.399), tramadol (0.338), morphine (0.314), and oxymorphone (0.173) (Figure S5c).

**Table 4:** League table showing the odds of TRIAL WITHDRAWAL with the treatment of various opioids.

**Tapentadol**									
0.58 (0.34–0.98)	**Oxycodone-Naloxone**								
0.59 (0.24–1.51)	1.02 (0.43–2.50)	**Fentanyl**							
0.51 (0.25–1.00)	0.87 (0.45–1.69)	0.86 (0.33–2.10)	**Hydro-morphone**						
0.50 (0.34–0.75)	0.87 (0.58–1.31)	0.85 (0.36–1.92)	0.99 (0.57–1.74)	**Oxycodone**					
0.41 (0.15–1.15)	0.71 (0.27–1.97)	0.70 (0.22–2.19)	0.81 (0.29–2.39)	0.82 (0.33–2.10)	**Bupre-norphine**				
0.41 (0.13–1.30)	0.71 (0.23–2.17)	0.69 (0.23–2.05)	0.81 (0.26–2.61)	0.81 (0.28–2.40)	0.99 (0.33–2.98)	**Methadone**			
0.36 (0.10–1.33)	0.63 (0.17–2.27)	0.62 (0.15–2.46)	0.72 (0.19–2.73)	0.72 (0.21–2.46)	0.88 (0.38–1.99)	0.89 (0.22–3.46)	**Tramadol**		
0.39 (0.21–0.75)	0.68 (0.38–1.22)	0.67 (0.33–1.29)	0.78 (0.41–1.50)	0.78 (0.47–1.31)	0.95 (0.35–2.57)	0.96 (0.34–2.69)	1.08 (0.30–3.96)	**Morphine**	
0.26 (0.08–0.83)	0.45 (0.14–1.43)	0.44 (0.11–1.69)	0.51 (0.15–1.74)	0.52 (0.17–1.53)	0.63 (0.15–2.58)	0.64 (0.14–2.91)	0.72 (0.14–3.72)	0.66 (0.20–2.18)	**Oxymorphone**

OR <1 means the treatment in top left is better.

### Patient satisfaction

Conventional meta-analysis revealed that oxycodone-naloxone treatment had significantly better patient satisfaction rate in comparison with other opioids (p < 0.0001). There was no significant difference in the patient satisfaction rate between either of tapentadol, oxycodone, fentanyl, hydromorphone, or buprenorphine and their comparator opioids (Table 1 and Figure S3d).

In NMA, total number of interventions were 8 which were carried out in 15 studies in 6,560 patients of which 3918 were satisfied with the treatment. Network, intervention, and direct comparison characteristics of this NMA are given in Tables S2–4. Oxycodone was the most investigated opioid (10 studies; compared with 6 different opioids) followed by morphine (5 studies) and tapentadol (4 studies each). This NMA revealed that highest patient satisfaction was associated with oxycodone-naloxone treatment, followed by fentanyl and tapentadol (Table 5). Probability for ranking top among the opioids was 0.89 for oxycodone-naloxone whereas the probability for ranking second highest was 0.743 for fentanyl (Figure S4d). SUCRA probabilities for various opioids were: oxycodone-naloxone (0.984), fentanyl (0.821), tapentadol (0.631), oxycodone (0.396), buprenorphine (0.371), morphine (0.309), hydromorphone (0.294), and tramadol (0.194) (Figure S5d).

**Table 5:** League table showing the odds ratios (OR) of various opioids in achieving **PATIENT SATISFACTION** in pain relief.

**Oxycodone-Naloxone**							
1.87 (0.67–5.31)	**Fentanyl**						
3.44 (2.04–6.32)	1.85 (0.65–5.39)	**Tapentadol**					
4.21 (2.76–7.03)	2.26 (0.84–6.33)	1.22 (0.89–1.71)	**Oxycodone**				
4.40 (2.09–10.11)	2.36 (0.72–7.86)	1.28 (0.62–2.63)	1.04 (0.55–1.98)	**Buprenorphine**			
4.51 (2.92–7.34)	2.42 (0.96–6.13)	1.32 (0.78–2.13)	1.07 (0.71–1.54)	1.03 (0.48–2.11)	**Morphine**		
4.60 (2.68–8.02)	2.45 (0.88–6.74)	1.34 (0.77–2.18)	1.09 (0.70–1.59)	1.05 (0.47–2.14)	1.02 (0.67–1.51)	**Hydromorphone**	
5.44 (2.15–15.13)	2.90 (0.79–11.07)	1.58 (0.64–3.99)	1.29 (0.55–3.06)	1.24 (0.70–2.23)	1.20 (0.49–3.15)	1.18 (0.48–3.17)	**Tramadol**

OR >1 means the treatment in top left is better.

Forest graphs of all NMAs are given in supporting information as Figure S5a–d.

## Discussion

Tapentadol treatment to patients with chronic pain conditions has been found to be associated with least incidence of overall adverse events as well as constipation and trial withdrawal rate in this mixed-treatment meta-analysis. Oxycodone-naloxone combination also exhibited good tolerability with lesser trial withdrawal rate. Patient satisfaction was found to be higher with oxycodone-naloxone followed by fentanyl and tapentadol. These results were in agreement with those achieved by the conventional meta-analyses.

Recently issued CDC guidelines for prescribing opioids for chronic pain recommend that opioids should be prescribed after risk assessment, and if risks outweigh benefits, a discontinuation strategy should be planned in advance. It is also recommended that lowest effective dosage should be prescribed earlier and then a re-assessment be made when considering increasing dosage equivalent to 50 mg/day morphine while avoiding concurrent opioids and benzodiazepines. A re-assessment of benefits and harms of continued opioid therapy should be made in less than every third month^16, 17^.

However, optimal and judicious use of opioids is still far away from the desired situation. A number of potentially serious adverse effects and aberrant behaviors such as opioid abuse, addiction, and diversion make it difficult for physicians to prescribe opioids liberally^56^. Therefore, not only the under-treatment of pain is recognized as a major problem for patients which has societal implications^6^ but also the opioids are underutilized drugs for chronic pain especially in elderly^57^ due to physician’s concerns about the uncertain efficacy, untoward side effects and addiction^58, 59^.

Benefits of opioids are generally limited by their tolerability which is reported to cause up to 31% discontinuation rate^14, 15^. Constipation, nausea, vomiting, dizziness, somnolence, pruritus, fatigue and anorexia are commonly observed in opioid treated patients. A review of 40 studies found that 25% of the opioid treated patients discontinued treatment due to an adverse event and in a subset of studies there was no significant difference in the incidence of opioid-related AEs in individuals of over or under 65 years of age without significant comorbidities^60^.

Tapentadol is a centrally acting synthetic opioid which not only act as opioid receptor agonist but also as a norepinephrine reuptake inhibitor^61, 62^. The controlled release formulation of tapentadol can provide analgesia for 12 hours. For chronic pain conditions, it is suitable because its µ-receptor activation is a secondary mechanism and therefore a relatively low degree of µ-receptor activation, in comparison with potent opioids, causes a slower onset of tolerance^62^. Review of head-to-head trials of tapentadol with other opioids such as oxycodone controlled release and oxycodone-naloxone has also shown that tapentadol is at least comparable in providing pain relief in moderate/severe chronic nociceptive as well as neuropathic pain conditions besides improving health status and quality of life^63, 64^. On the other hand, incidences of common opioid-related adverse events such as nausea and/or vomiting, and constipation as well as discontinuation rate are found to be significantly lower in the tapentadol treated groups in comparison with other opioid treated groups^64^.

Oxycodone-naloxone combination was developed basically to reduce the incidence of opioid-induced constipation. In a typical tablet, naloxone is added as an opioid receptor antagonist to prevent oxycodone activity in the gut while its prolonged release and extensive metabolism in the liver leads to low bioavailability (2%) which remains insufficient to antagonize oxycodone action centrally^48, 65^. In the present study, oxycodone-naloxone combination has been found to attain second rank after tapentadol with regards to its benefits in low incidence of adverse events, constipation and trial withdrawal. However, in patient satisfaction, it attained first rank. Beyond the present study too, it has been reported that oxycodone-naloxone combination is safe and well tolerable and is superior in effectiveness to oxycodone^66–68^.

Opioids therapy for chronic pain is challenging due to clinical, adverse, and end-organ effects as well as due to their potential interactions with other medications. Although, tolerability is a major issue with opioid therapy, nevertheless, opioid analgesia provides good relief with manageable adverse effects to selected patients suffering from chronic pain. Centrally acting analgesics with a dual mechanism of action offer therapies with improved tolerability. Tapentadol and oxycodone-naloxone appear to advance opioid therapy in this direction. However, a better patient-centered care in opioid therapy for chronic pain is more important keeping in view the high inter-individual variability in responsiveness to opioids and an earlier start of medication can reduce the progression of pain.

There can be several factors to affect the outcomes of this mixed-treatment meta-analysis including route and mode of drug administration, ethnicity and geographical distribution of patients, treatment duration, and history of opioid use. Modes of drug administration in the present study varied across the included studies and for some modes as well as routes of administration, less data (studies) were available to classify each mode as a separate regimen, therefore, impact of mode of administration will need to be further studied in larger datasets. Although, recent trend is towards more concentrated dosage and controlled-release preparations with oral and transdermal opioid formulations but other factors may also play a role in determining the suitability of a drug delivery system e.g. the patient’s preferences, delivery efficiency, potential complications of a system, and financial cost of formulation or device^69^.

Ethnic differences in response to pain management are reported by many researchers. Besides, inconsistencies in pain medication prescription, different ethnic groups were reported to use pain medications in different quantities even when equal access e.g. with patient-controlled analgesia was ensured^70^. History of opioid use may be associated with inadequacy of opioid treatment and or efficacy and may have potential for consequences such as illicit drug use, misuse of prescription drugs, psychiatric distress, etc.^71^. To evaluate the effects of these factors on overall outcomes, a relatively larger dataset is required that can be used to identify the prognostic factors to help decision-making in the clinical use of opioids and in selecting subgroup of patients for a particular opioid therapy.

Some other limitations are also needed to be account for while interpreting the outcomes observed herein. Number of studies investigating some types of opioids were much less, especially for methadone and oxymorphone. Many of the included studies also involved open-label designs which might had introduced a risk of bias because patients or investigators might had taken/prescribed concomitant treatments to enhance efficacy or to improve tolerability based on their knowledge and beliefs of treatment allocation^66^. However, it has been opined that potential benefits of an open-label design may outweigh those of a blinded design in this population^43^ and not blinding the study could be sometimes intentionally directed by the need to mimic a daily clinical trite where therapeutic flexibility is needed^11^. This may also be attributed to the difficulties in performing controlled trials in this population. Additionally, trial duration was relatively smaller in some studies which necessitates the assessment of trial duration on the overall effectiveness.

## Conclusion

Tapentadol treatment in chronic pain conditions is associated with least incidence of overall adverse events and constipation, as well as with least trial withdrawal rate. Oxycodone-naloxone combination also share superiority with tapentadol in providing better tolerability and less trial withdrawal rate. Patient satisfaction was found to be higher with oxycodone-naloxone followed by fentanyl and tapentadol. Conventional meta-analyses also endorsed these findings.

## Electronic supplementary material

Supporting information
