# Supplementary material for: Tolerability of Opioid Analgesia for Chronic Pain: A Network Meta-Analysis
Source: Sci Rep. 2017 May 17;7:1995. doi: 10.1038/s41598-017-02209-x (PMC5435686; doi:10.1038/s41598-017-02209-x)
Supplement: Supplementary file 1 — Supporting information [file 41598_2017_2209_MOESM1_ESM.doc]

**Tolerability of Opioid Analgesia for Chronic Pain: A Network Meta-Analysis**

Short Title: Tolerability of Opioids for chronic pain

Zengdong Meng^1^, Jing Yu ^4^, Michael Acuff ^5^, Chong Luo^1^, Sanrong Wang^2,3^, Lehua Yu^2,3^, Rongzhong Huang^2,3*^

^1^ Department of Orthopaedics, First People’s Hospital of YunNan Province, YunNan, P. R. China

^2^ Department of Rehabilitation Medicine, The second Affiliated Hospital of Chongqing Medical University, Chongqing, P. R. China

^3^ Department of Pain Medicine, The second Affiliated Hospital of Chongqing Medical University, Chongqing, P. R. China

^4^ Department of Preventive Medicine, Keck School of Medicine, University of Southern California, Los Angeles, CA

^5^ Rusk Rehabilitation Center, University of Missouri School of Medicine

***Corresponding address:**

Dr. Rongzhong Huang

Department of Rehabilitation Medicine

The Second Affiliated Hospital of Chongqing Medical University

No. 76 Linjiang Road, Chongqing 400010, China

Tel: +86-18523457722

E-mail: [rzhuang@live.com](mailto:rzhuang@live.com)

**Supporting information**

Tolerability of Opioid Analgesia for Chronic Pain: A Network Meta-Analysis

| **Table S1: Risk of Bias Assessment of the included studies** | | | | | | | |
| --- | --- | --- | --- | --- | --- | --- | --- |
| **Study** | **Other bias** | **Selective reporting** | **Incomplete outcome data** | **Blinding of outcome assessment** | **Blinding of participants /personnel** | **Allocation concealment** | **Random sequence generator** |
| Ahmedzai 2011 | U | L | L | L | L | L | L |
| Allan 2001 | L | L | L | H | H | H | L |
| Allan 2005 | L | L | L | H | H | H | L |
| Baron 2015 | L | L | L | H | H | H | L |
| Binsfeld 2010 | L | L | L | H | H | H | L |
| Etropolski 2011 | L | L | L | L | L | L | L |
| Etropolski 2014 | L | L | L | H | H | H | L |
| Hale 2007 | L | L | L | H | H | H | L |
| Hale 2009 | L | L | L | L | L | L | L |
| Hanna 2008 | L | L | L | L | L | L | L |
| Hanna 2009 | L | L | L | H | H | H | L |
| Hartick 2009 | L | L | L | L | L | L | L |
| Imanaka 2013 | L | L | L | L | L | L | L |
| Karlsson 2009 | L | L | L | H | H | H | L |
| Kavanagh 2012 | U | U | L | L | L | L | L |
| Lange 2010 | L | L | L | L | L | L | L |
| Leng 2015 | L | L | L | L | L | L | L |
| Lowenstein 2009 | U | L | L | L | L | L | L |
| Matsumoto 2005 | U | L | L | L | L | L | L |
| Mercadante 2008 | U | H | L | H | H | H | L |
| Neumann 2013 | U | H | L | H | H | H | L |
| Nicholson 2006 | U | L | L | H | H | H | L |
| Richarz 2013 | U | L | L | H | H | H | L |
| Steiner 2011 | L | L | L | L | L | L | L |
| Ueberal 2015 | L | L | L | L | H | L | L |
| Ueberal 2016 | L | L | L | L | H | L | L |
| van Seventer 2003 | L | L | L | H | H | H | L |
| Vondrackova 2008 | U | L | L | L | L | L | L |
| Wallace 2007 | U | H | L | H | H | H | L |
| Webster 2006 | U | L | L | L | L | L | L |
| Webster 2013 | U | H | L | L | L | L | L |
| Yu 2014 | U | H | L | L | L | L | L |
| Legends: H, high bias; L, low bias; U, unclear bias | | | | | | | |

Table S2a NMA characteristics - Data summary of the network meta-analysis of the odds ratios between various opioids in the incidence of overall **ADVERSE EVENTS**

| **Network Characteristics** | | | | | |  | |  | | | |  | |  | |  | |  | |
| --- | --- | --- | --- | --- | --- | --- | --- | --- | --- | --- | --- | --- | --- | --- | --- | --- | --- | --- | --- |
| **Characteristic** | | | | | | **Number** | |  | | | |  | |  | |  | |  | |
| **Number of Interventions** | | | | | | 9 | |  | | | |  | |  | |  | |  | |
| **Number of Studies** | | | | | | 25 | |  | | | |  | |  | |  | |  | |
| **Total Number of Patient-weeks in Network** | | | | | | 280,292 | |  | | | |  | |  | |  | |  | |
| **Total Number of Events in Network** | | | | | | 9,989 | |  | | | |  | |  | |  | |  | |
| **Total Possible Pairwise Comparisons** | | | | | | 36 | |  | | | |  | |  | |  | |  | |
| **Total Number Pairwise Comparisons with Direct Data** | | | | | | 12 | |  | | | |  | |  | |  | |  | |
| **Number of Two-arm Studies** | | | | | | 24 | |  | | | |  | |  | |  | |  | |
| **Number of Multi-Arms Studies** | | | | | | 1 | |  | | | |  | |  | |  | |  | |
| **Number of Studies with No Zero Events** | | | | | | 25 | |  | | | |  | |  | |  | |  | |
| **Number of Studies With at least One Zero Event** | | | | | | 0 | |  | | | |  | |  | |  | |  | |
| **Number of Studies with All Zero Events** | | | | | | 0 | |  | | | |  | |  | |  | |  | |
| **Intervention Characteristics** | | | |  | | | | |  | |  |  |  | | | | | | |
| **Treatment** | **# Studies** | **# Events** | | **# Patient-weeks** | | | | | **Aggregate Rate** | | **Min. Rate** | **Max. Rate** |  | | | | | | |
| **Oxycodone** | 17 | 2734 | | 95912 | | | | | 0.0285 | | 0.0126 | 0.6603 |  | | | | | | |
| **Oxycodone-Naloxone** | 5 | 601 | | 8,600 | | | | | 0.0699 | | 0.0260 | 0.7337 |  | | | | | | |
| **Tapentadol** | 6 | 1970 | | 105,898 | | | | | 0.0186 | | 0.0091 | 0.4762 |  | | | | | | |
| **Morphine** | 7 | 1623 | | 24258 | | | | | 0.0669 | | 0.0267 | 0.7558 |  | | | | | | |
| **Fentanyl** | 3 | 1174 | | 19498 | | | | | 0.0602 | | 0.0578 | 0.2276 |  | | | | | | |
| **Hydromorphone** | 7 | 711 | | 19438 | | | | | 0.0366 | | 0.0129 | 0.7403 |  | | | | | | |
| **Buprenorphine** | 3 | 579 | | 4488 | | | | | 0.1290 | | 0.0643 | 0.3087 |  | | | | | | |
| **Tramadol** | 2 | 335 | | 1724 | | | | | 0.1943 | | 0.1646 | 0.2484 |  | | | | | | |
| **Oxymorphone** | 1 | 262 | | 476 | | | | | 0.5504 | | 0.5504 | 0.5504 |  | | | | | | |
| **Direct Comparison Characteristics** | | | | | | |  | | |  | | | | |  | |  | |  |
| **Comparison** | | | **# Studies** | | **# Patients** | | **# Events** | | |  | | | | |  | |  | |  |
| **Oxycodone vs. Oxycodone-Naloxone** | | | 4 | | 14,980 | | 992 | | |  | | | | |  | |  | |  |
| **Morphine vs. Fentanyl** | | | 2 | | 38,156 | | 2,355 | | |  | | | | |  | |  | |  |
| **Oxycodone-Naloxone vs. Tapentadol** | | | 1 | | 2,322 | | 207 | | |  | | | | |  | |  | |  |
| **Oxycodone vs. Hydromorphone** | | | 4 | | 33,876 | | 767 | | |  | | | | |  | |  | |  |
| **Oxycodone vs. Tapentadol** | | | 5 | | 171,540 | | 3,281 | | |  | | | | |  | |  | |  |
| **Morphine vs. Hydromorphone** | | | 3 | | 4,262 | | 641 | | |  | | | | |  | |  | |  |
| **Buprenorphine vs. Tramadol** | | | 1 | | 3,584 | | 745 | | |  | | | | |  | |  | |  |
| **Oxycodone vs. Oxymorphone** | | | 1 | | 976 | | 492 | | |  | | | | |  | |  | |  |
| **Oxycodone vs. Morphine** | | | 2 | | 6,300 | | 204 | | |  | | | | |  | |  | |  |
| **Oxycodone vs. Buprenorphine** | | | 1 | | 5,268 | | 329 | | |  | | | | |  | |  | |  |
| **Oxycodone-Naloxone vs. Morphine** | | | 1 | | 3,624 | | 120 | | |  | | | | |  | |  | |  |
| **Oxycodone vs. Fentanyl** | | | 1 | | 840 | | 45 | | |  | | | | |  | |  | |  |

Table S2b NMA characteristics - Data summary of the network meta-analysis of the odds ratios between various opioids in the incidence of **CONSTIPATION**

| **Network Characteristics** | | | | |  | |  | | | | |
| --- | --- | --- | --- | --- | --- | --- | --- | --- | --- | --- | --- |
| **Characteristic** | | | | | **Number** | |  | | | | |
| **Number of Interventions** | | | | | 9 | |  | | | | |
| **Number of Studies** | | | | | 25 | |  | | | | |
| **Total Number of Patient-weeks in Network** | | | | | 286,864 | |  | | | | |
| **Total Number of Events in Network** | | | | | 2,831 | |  | | | | |
| **Total Possible Pairwise Comparisons** | | | | | 36 | |  | | | | |
| **Total Number Pairwise Comparisons with Direct Data** | | | | | 11 | |  | | | | |
| **Number of Two-arm Studies** | | | | | 23 | |  | | | | |
| **Number of Multi-Arms Studies** | | | | | 2 | |  | | | | |
| **Number of Studies with No Zero Events** | | | | | 25 | |  | | | | |
| **Number of Studies With At least One Zero Event** | | | | | 0 | |  | | | | |
| **Number of Studies with All Zero Events** | | | | | 0 | |  | | | | |
| **Intervention Characteristics** | | | |  | |  | | |  | |  |
| **Treatment** | **# Studies** | **# Events** | | **# Patients** | | **Aggregate Rate** | | | **Min. Rate** | | **Max. Rate** |
| **Oxycodone** | 17 | 1102 | | 97654 | | 0.0113 | | | 0.0028 | | 0.1744 |
| **Oxycodone-Naloxone** | 7 | 192 | | 13,364 | | 0.0144 | | | 0.0006 | | 0.0330 |
| **Tapentadol** | 6 | 565 | | 105,898 | | 0.0053 | | | 0.0038 | | 0.0759 |
| **Morphine** | 8 | 482 | | 27858 | | 0.0173 | | | 0.0052 | | 0.1105 |
| **Fentanyl** | 2 | 188 | | 19084 | | 0.0099 | | | 0.0094 | | 0.0448 |
| **Hydromorphone** | 6 | 203 | | 16318 | | 0.0124 | | | 0.0055 | | 0.1948 |
| **Buprenorphine** | 3 | 36 | | 4488 | | 0.0080 | | | 0.0053 | | 0.0178 |
| **Tramadol** | 2 | 15 | | 1724 | | 0.0087 | | | 0.0082 | | 0.0090 |
| **Oxymorphone** | 1 | 48 | | 476 | | 0.1008 | | | 0.1008 | | 0.1008 |
| **Direct Comparison Characteristics** | | | | | | | |  | |  | |
| **Comparison** | | | **# Studies** | | **# Patients** | | | **# Events** | |  | |
| **Oxycodone vs. Oxycodone-Naloxone** | | | 6 | | 24,616 | | | 456 | |  | |
| **Morphine vs. Fentanyl** | | | 2 | | 38,156 | | | 426 | |  | |
| **Oxycodone-Naloxone vs. Tapentadol** | | | 1 | | 2,322 | | | 53 | |  | |
| **Oxycodone vs. Hydromorphone** | | | 3 | | 28,052 | | | 264 | |  | |
| **Oxycodone vs. Tapentadol** | | | 5 | | 171,540 | | | 1,158 | |  | |
| **Morphine vs. Hydromorphone** | | | 3 | | 4,262 | | | 105 | |  | |
| **Buprenorphine vs. Tramadol** | | | 1 | | 3,584 | | | 37 | |  | |
| **Oxycodone vs. Oxymorphone** | | | 1 | | 976 | | | 93 | |  | |
| **Oxycodone vs. Morphine** | | | 3 | | 13,500 | | | 399 | |  | |
| **Oxycodone vs. Buprenorphine** | | | 1 | | 5,268 | | | 28 | |  | |
| **Oxycodone-Naloxone vs. Morphine** | | | 2 | | 10,836 | | | 292 | |  | |

Table S2c: NMA characteristics - Data summary of the network meta-analysis of the odds ratios between various opioids in **TRIAL WITHDRAWAL RATE**

| **Network Characteristics** | | | | | |  | | |  | | | |
| --- | --- | --- | --- | --- | --- | --- | --- | --- | --- | --- | --- | --- |
| **Characteristic** | | | | | | **Number** | | |  | | | |
| **Number of Interventions** | | | | | | 10 | | |  | | | |
| **Number of Studies** | | | | | | 27 | | |  | | | |
| **Total Number of Patients in Network** | | | | | | 12,207 | | |  | | | |
| **Total Number of Events in Network** | | | | | | 4,822 | | |  | | | |
| **Total Possible Pairwise Comparisons** | | | | | | 45 | | |  | | | |
| **Total Number Pairwise Comparisons With Direct Data** | | | | | | 14 | | |  | | | |
| **Number of Two-arm Studies** | | | | | | 24 | | |  | | | |
| **Number of Multi-Arms Studies** | | | | | | 3 | | |  | | | |
| **Number of Studies With No Zero Events** | | | | | | 27 | | |  | | | |
| **Number of Studies With At Least One Zero Event** | | | | | | 0 | | |  | | | |
| **Number of Studies with All Zero Events** | | | | | | 0 | | |  | | | |
| **Intervention Characteristics** | | | |  | | |  | | |  | |  |
| **Treatment** | **# Studies** | **# Events** | | **# Patients** | | | **Aggregate Rate** | | | **Min. Rate** | | **Max. Rate** |
| **Oxycodone** | 18 | 1787 | | 4100 | | | 0.4359 | | | 0.1192 | | 0.6225 |
| **Oxycodone-Naloxone** | 7 | 389 | | 1,317 | | | 0.2954 | | | 0.1169 | | 0.6250 |
| **Tapentadol** | 7 | 1407 | | 3,875 | | | 0.3631 | | | 0.0619 | | 0.4840 |
| **Morphine** | 8 | 472 | | 1053 | | | 0.4482 | | | 0.2208 | | 0.8182 |
| **Fentanyl** | 3 | 206 | | 441 | | | 0.4671 | | | 0.2687 | | 0.5237 |
| **Hydromorphone** | 5 | 271 | | 576 | | | 0.4705 | | | 0.1512 | | 0.8857 |
| **Buprenorphine** | 4 | 140 | | 455 | | | 0.3077 | | | 0.2029 | | 0.5000 |
| **Tramadol** | 2 | 54 | | 205 | | | 0.2634 | | | 0.2374 | | 0.3182 |
| **Oxymorphone** | 1 | 68 | | 121 | | | 0.5620 | | | 0.5620 | | 0.5620 |
| **Methadone** | 2 | 28 | | 64 | | | 0.4375 | | | 0.3611 | | 0.5357 |
| **Direct Comparison Characteristics** | | | | | | | |  | | |  | |
| **Comparison** | | | **# Studies** | | **# Patients** | | | **# Events** | | |  | |
| **Oxycodone vs. Oxycodone-Naloxone** | | | 6 | | 2,384 | | | 681 | | |  | |
| **Morphine vs. Fentanyl** | | | 3 | | 883 | | | 420 | | |  | |
| **Oxycodone-Naloxone vs. Tapentadol** | | | 1 | | 258 | | | 124 | | |  | |
| **Oxycodone vs. Hydromorphone** | | | 3 | | 904 | | | 459 | | |  | |
| **Oxycodone vs. Tapentadol** | | | 6 | | 5,802 | | | 2,410 | | |  | |
| **Morphine vs. Hydromorphone** | | | 2 | | 231 | | | 88 | | |  | |
| **Buprenorphine vs. Tramadol** | | | 1 | | 415 | | | 108 | | |  | |
| **Oxycodone vs. Oxymorphone** | | | 1 | | 246 | | | 118 | | |  | |
| **Morphine vs. Methadone** | | | 1 | | 72 | | | 27 | | |  | |
| **Fentanyl vs. Methadone** | | | 1 | | 72 | | | 24 | | |  | |
| **Buprenorphine vs. Methadone** | | | 1 | | 54 | | | 28 | | |  | |
| **Oxycodone vs. Morphine** | | | 3 | | 1,006 | | | 415 | | |  | |
| **Oxycodone vs. Buprenorphine** | | | 1 | | 439 | | | 134 | | |  | |
| **Oxycodone-Naloxone vs. Morphine** | | | 2 | | 903 | | | 311 | | |  | |

Table S2d: NMA characteristics - Data summary of the network meta-analysis of the odds ratios between various opioids in **PATIENTS’ SATISFACTION**

| **Network Characteristics** | | | | |  | | |  | | | | | |
| --- | --- | --- | --- | --- | --- | --- | --- | --- | --- | --- | --- | --- | --- |
| **Characteristic** | | | | | **Number** | | |  | | | | | |
| **Number of Interventions** | | | | | 8 | | |  | | | | | |
| **Number of Studies** | | | | | 15 | | |  | | | | | |
| **Total Number of Patients in Network** | | | | | 6,560 | | |  | | | | | |
| **Total Number of Events in Network** | | | | | 3,918 | | |  | | | | | |
| **Total Possible Pairwise Comparisons** | | | | | 28 | | |  | | | | | |
| **Total Number Pairwise Comparisons With Direct Data** | | | | | 9 | | |  | | | | | |
| **Number of Two-arm Studies** | | | | | 13 | | |  | | | | | |
| **Number of Multi-Arms Studies** | | | | | 2 | | |  | | | | | |
| **Number of Studies With No Zero Events** | | | | | 15 | | |  | | | | | |
| **Number of Studies With At Least One Zero Event** | | | | | 0 | | |  | | | | | |
| **Number of Studies with All Zero Events** | | | | | 0 | | |  | | | | | |
| **Intervention Characteristics** | | | |  | | |  | | |  | |  | |
| **Treatment** | | **# Studies** | **# Events** | **# Patients** | | | **Aggregate Rate** | | | **Min. Rate** | | **Max. Rate** | |
| **Oxycodone** | | 10 | 1131 | 2069 | | | 0.5466 | | | 0.4125 | | 0.8654 | |
| **Oxycodone-Naloxone** | | 2 | 344 | 445 | | | 0.7730 | | | 0.7276 | | 0.8681 | |
| **Tapentadol** | | 4 | 895 | 1,709 | | | 0.5237 | | | 0.4198 | | 0.8968 | |
| **Morphine** | | 5 | 581 | 958 | | | 0.6065 | | | 0.4000 | | 0.8023 | |
| **Fentanyl** | | 1 | 52 | 67 | | | 0.7761 | | | 0.7761 | | 0.7761 | |
| **Hydromorphone** | | 5 | 578 | 756 | | | 0.7646 | | | 0.6719 | | 0.9167 | |
| **Buprenorphine** | | 3 | 232 | 389 | | | 0.5964 | | | 0.5388 | | 0.6863 | |
| **Tramadol** | | 2 | 105 | 167 | | | 0.6287 | | | 0.5323 | | 0.6857 | |
|  | **Direct Comparison Characteristics** | | | | | | | | | |  | |  |
|  | **Comparison** | | | | | **# Studies** | | | **# Patients** | | **# Events** | |  |
|  | **Oxycodone vs. Hydromorphone** | | | | | 3 | | | 636 | | 485 | |  |
|  | **Oxycodone vs. Tapentadol** | | | | | 4 | | | 2,800 | | 1,457 | |  |
|  | **Morphine vs. Hydromorphone** | | | | | 2 | | | 863 | | 670 | |  |
|  | **Buprenorphine vs. Tramadol** | | | | | 1 | | | 337 | | 219 | |  |
|  | **Oxycodone vs. Buprenorphine** | | | | | 1 | | | 439 | | 239 | |  |
|  | **Oxycodone vs. Oxycodone-Naloxone** | | | | | 2 | | | 896 | | 564 | |  |
|  | **Oxycodone vs. Morphine** | | | | | 2 | | | 909 | | 414 | |  |
|  | **Oxycodone-Naloxone vs. Morphine** | | | | | 2 | | | 903 | | 538 | |  |
|  | **Morphine vs. Fentanyl** | | | | | 1 | | | 131 | | 90 | |  |

Figure S1a: Network diagrams of the incidence of adverse events NMA. Solid circle size represents the number of trials of a particular opioid drug and joining lines representing the head to head trials and thickness of lines indicate strength of comparisons.

Figure S1b: Network diagrams of the incidence of constipation NMA. Solid circle size represents the number of trials of a particular opioid drug and joining lines representing the head to head trials and thickness of lines indicate strength of comparisons.

Figure S1c: Network diagrams of the trial withdrawal rate NMA. Solid circle size represents the number of trials of a particular opioid drug and joining lines representing the head to head trials and thickness of lines indicate strength of comparisons.

Figure S1d: Network diagrams of the Patients satisfaction NMA. Solid circle size represents the number of trials of a particular opioid drug and joining lines representing the head to head trials and thickness of lines indicate strength of comparisons.

| 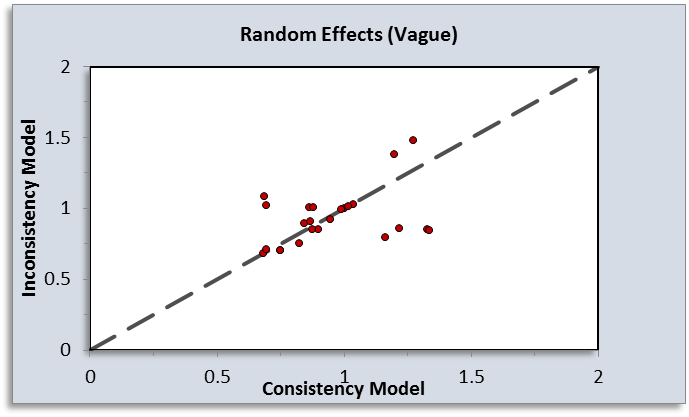 | 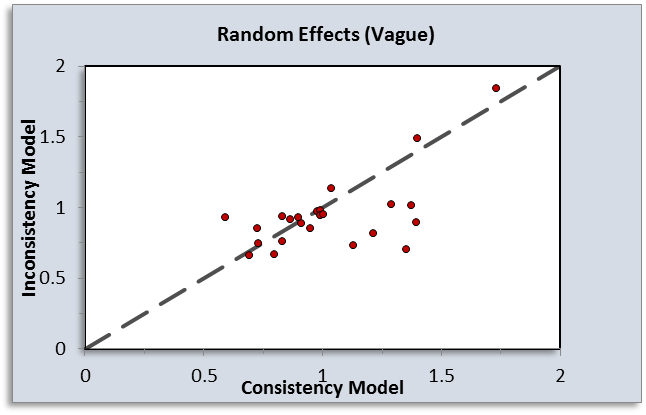 |
| --- | --- |
| **a** | **b** |
| 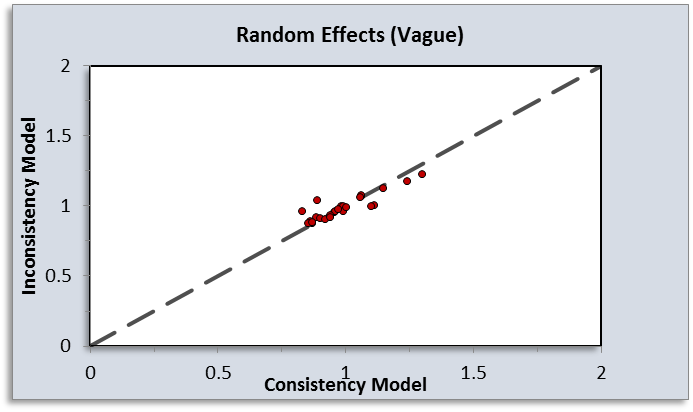 | 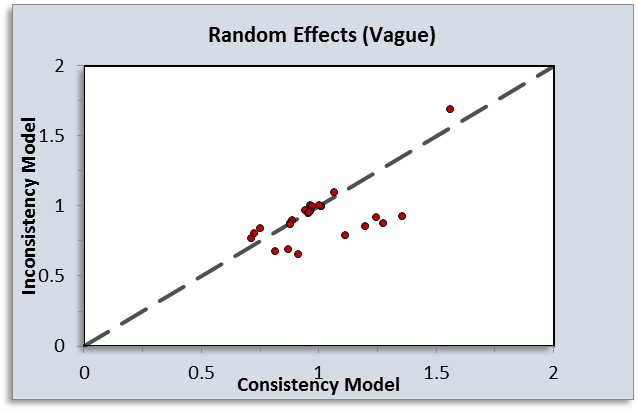 |
| **c** | **d** |

Figure S2: Plot showing the posterior mean deviance of the individual data points in the inconsistency model against their posterior mean deviance in the consistency model; a) incidence of adverse events NMA, b) incidence of constipation NMA, c) trial withdrawal rate, and d) patient satisfaction with treatment NMA. No significant inconsistency between direct and indirect evidence was observed for any study endpoint.


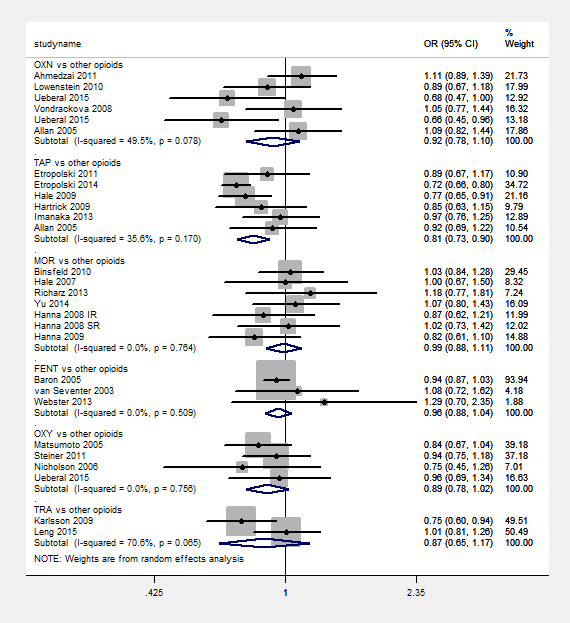


Figure S3a: A forest graph of the conventional meta-analysis showing the odds of a particular opioid in the incidence of **ADVERSE EVENTS** in comparison with other opioids


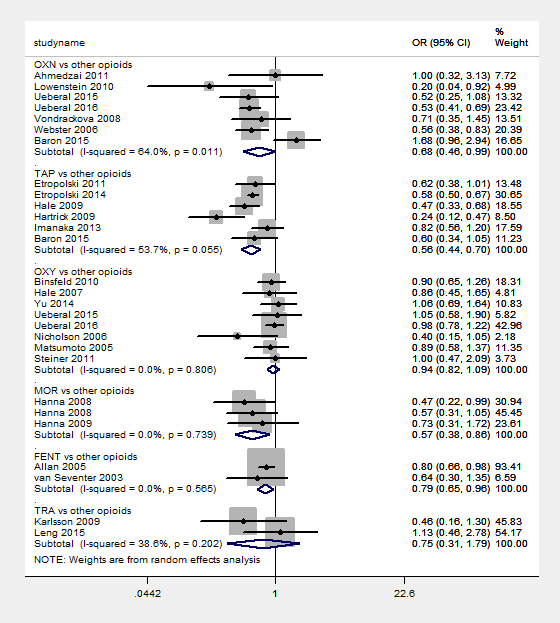


Figure S3b: A forest graph of the conventional meta-analysis showing the odds of a particular opioid in causing **CONSTIPATION** in comparison with other opioids


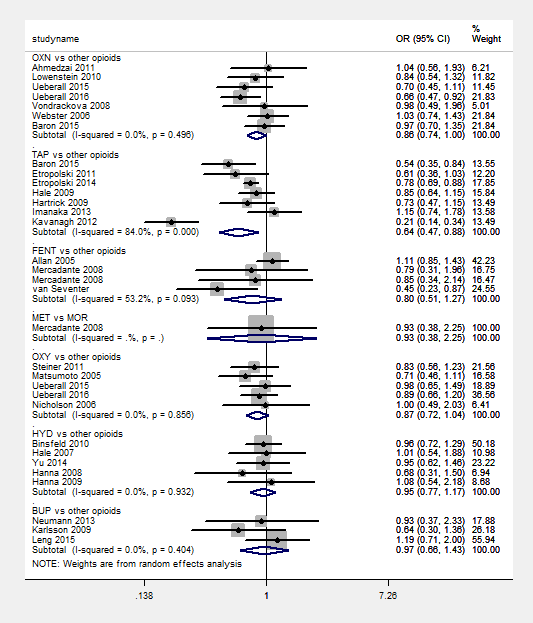


Figure S3c: A forest graph of the conventional meta-analysis showing the odds of a particular opioid in leading to **TRIAL WITHDRAWAL** in comparison with other opioids


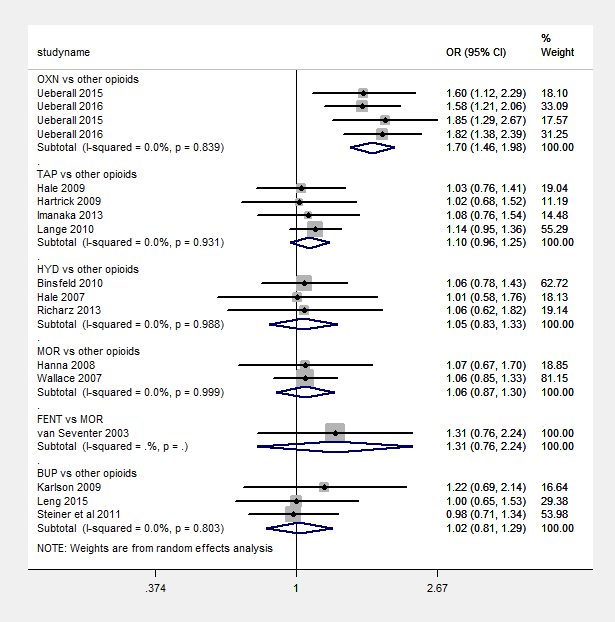


Figure S3d: A forest graph of the conventional meta-analysis showing the odds ratios of the **PATIENT SATISFACTION** in comparison with other opioids


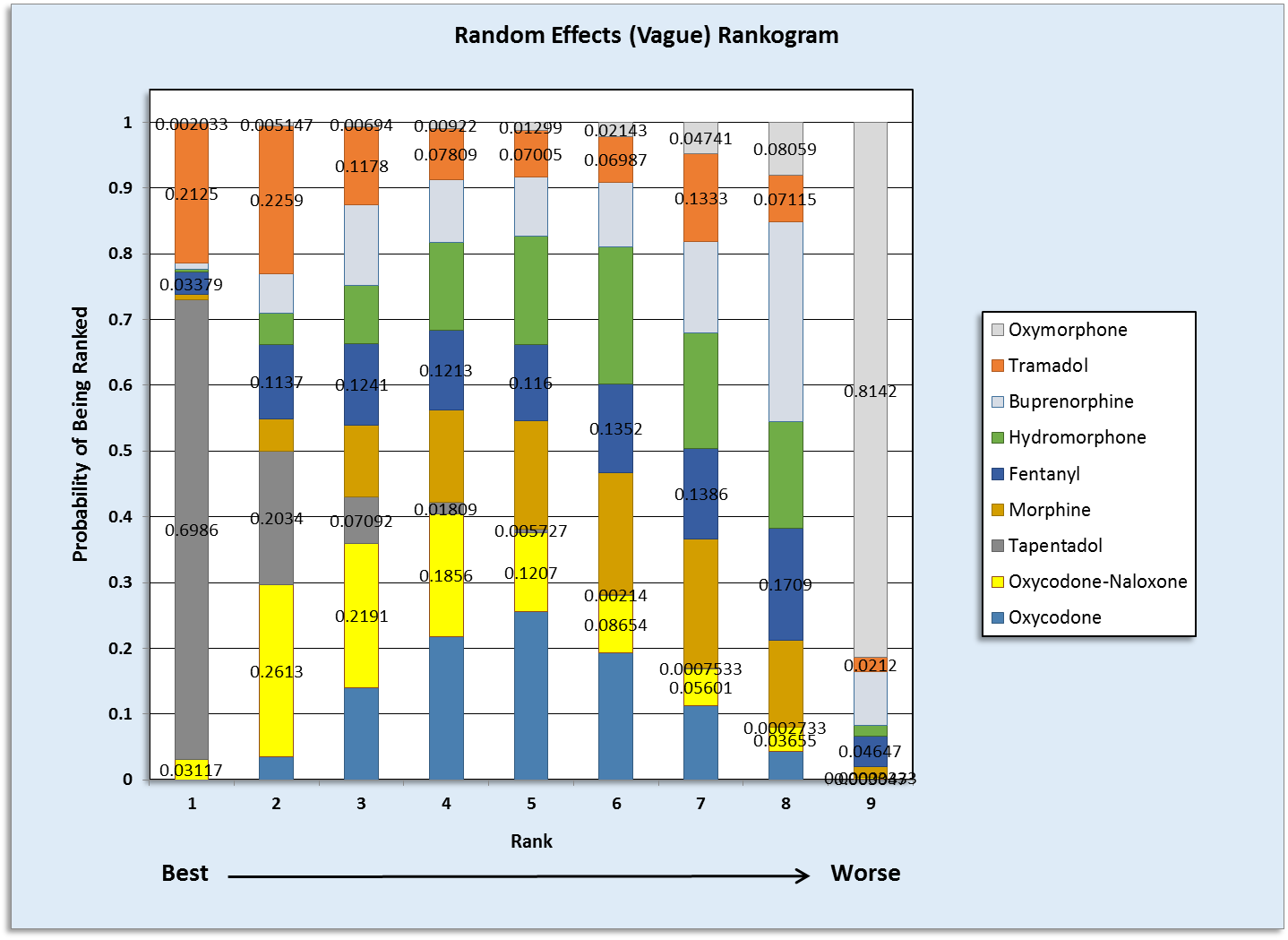


Figure S4a: Rankogram of the network meta-analysis of the odds ratios between various opioids in the incidence of overall **ADVERSE EVENTS** showing the probability any opioid of being ranked from best to worst


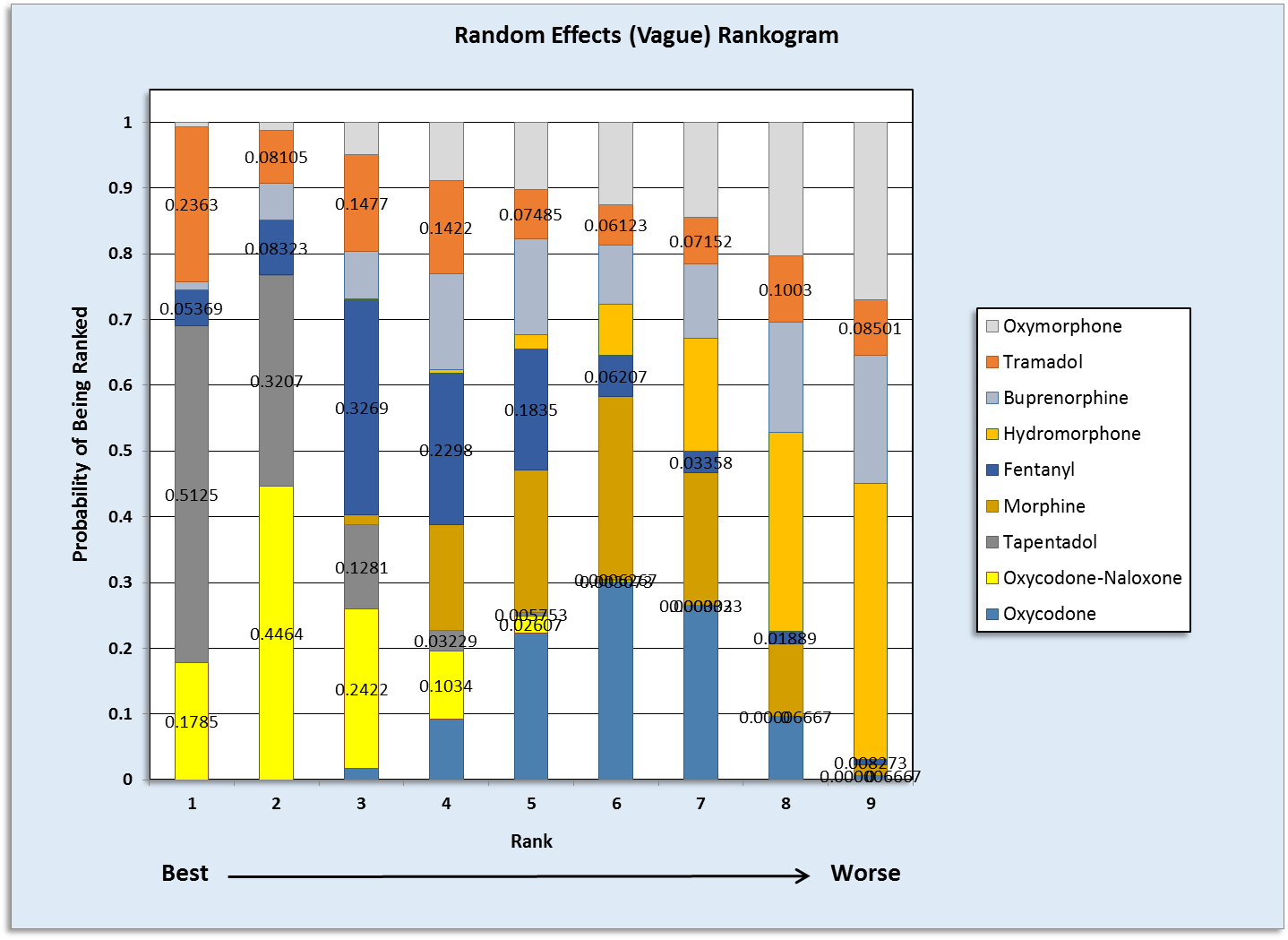


Figure S4b: Rankogram of the network meta-analysis of the odds ratios between various opioids in the incidence of **CONSTIPATION** showing the probability any opioid of being ranked from best to worst


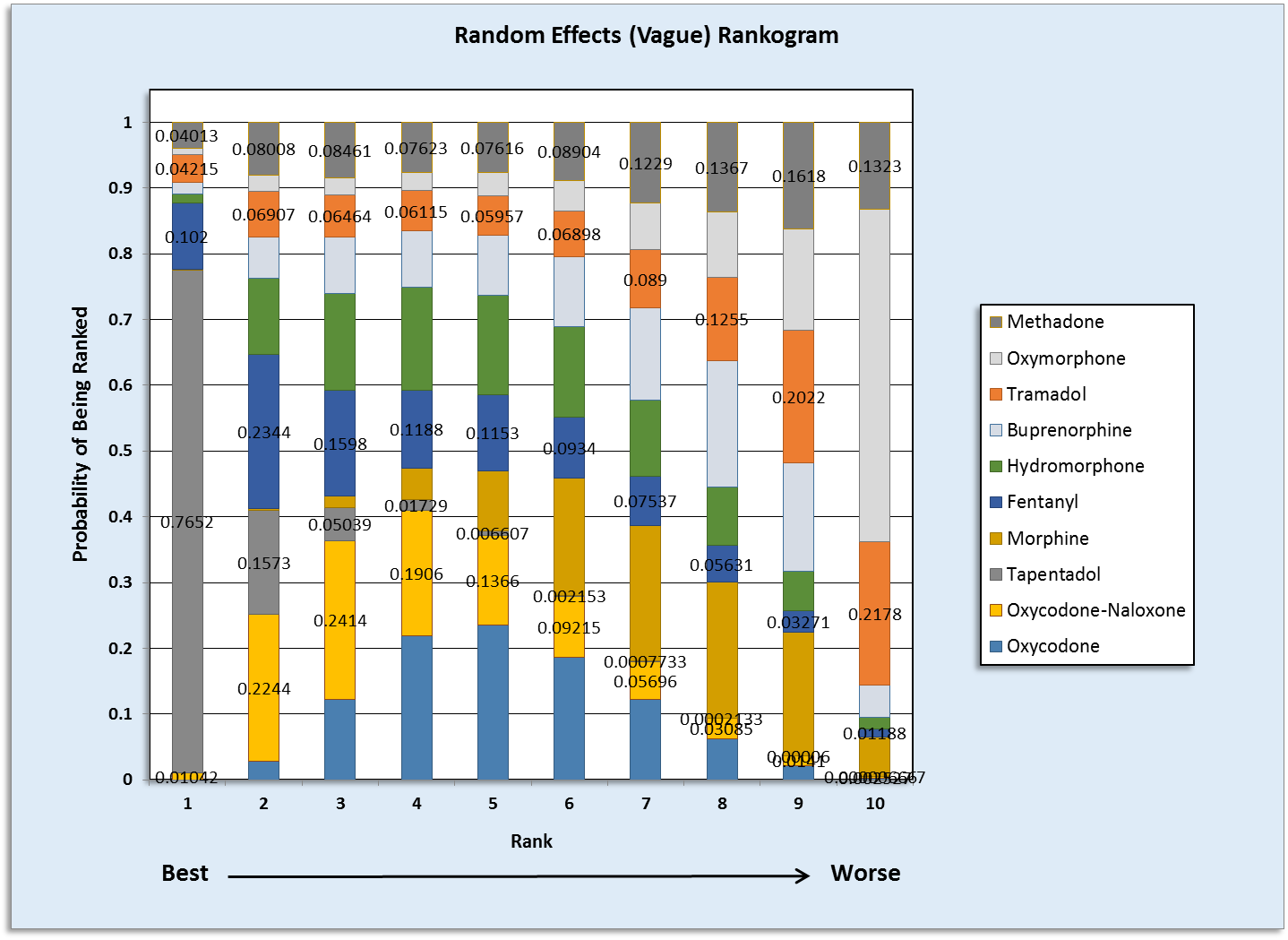


Figure S4c: Rankogram of the network meta-analysis of the odds ratios between various opioids in trial’s **DISCONTINUATION RATE** showing the probability any opioid of being ranked from best to worst


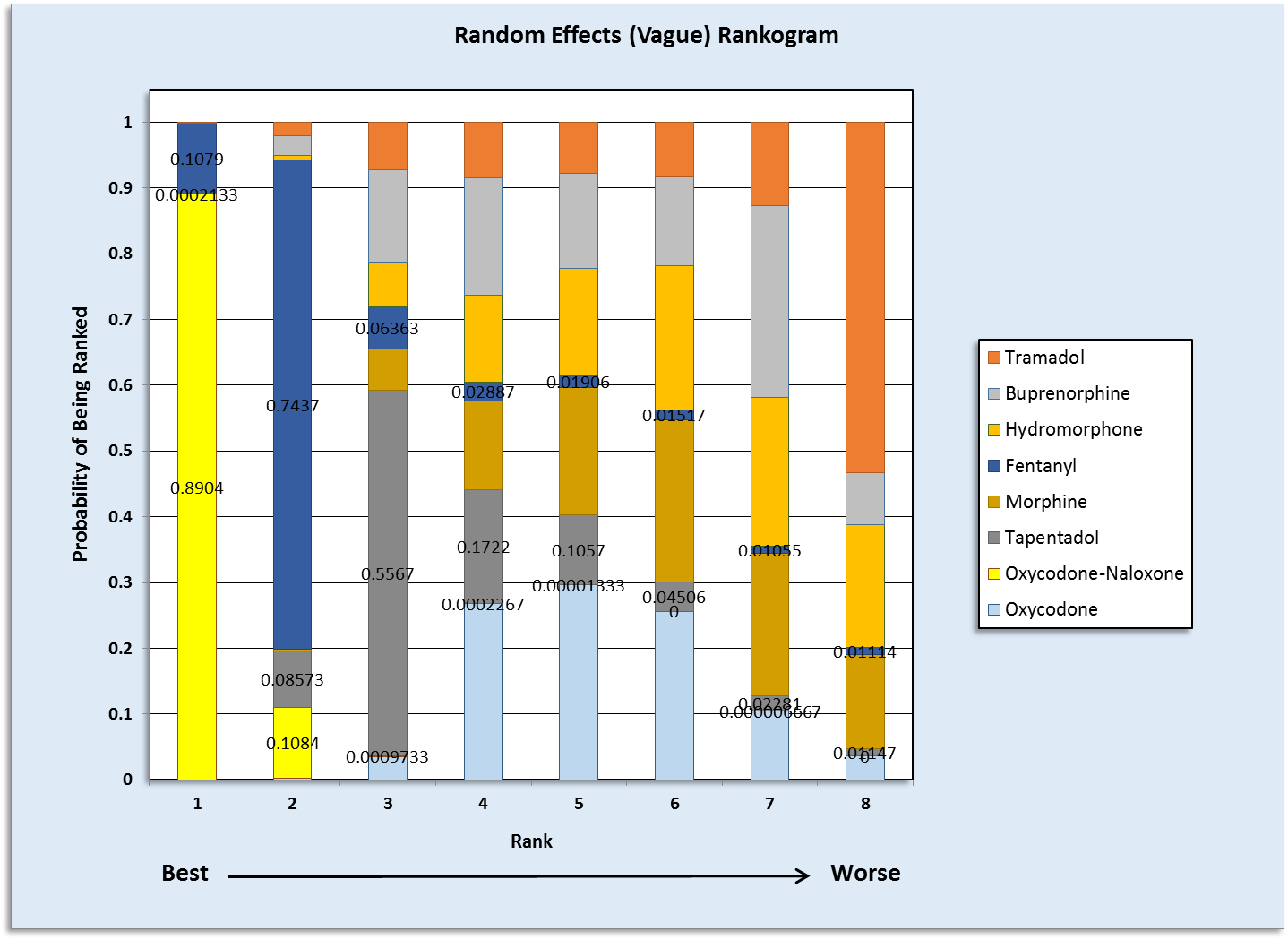


Figure S4d: Rankogram of the network meta-analysis of the odds ratios between various opioids in **PATIENTS SATISFACTION** showing the probability any opioid of being ranked from best to worst

| 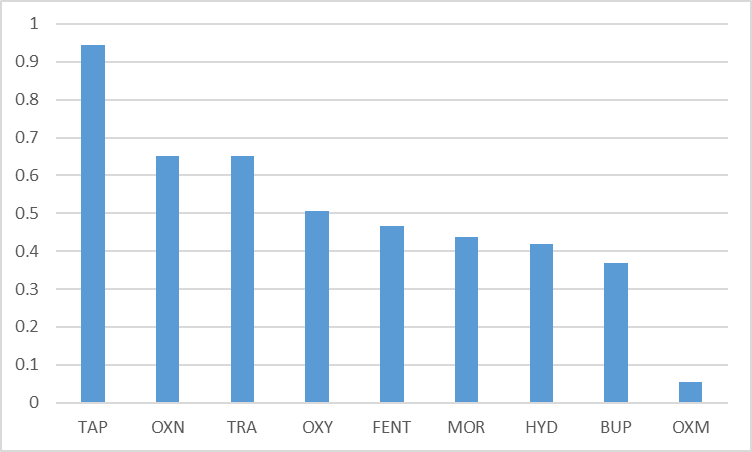 | 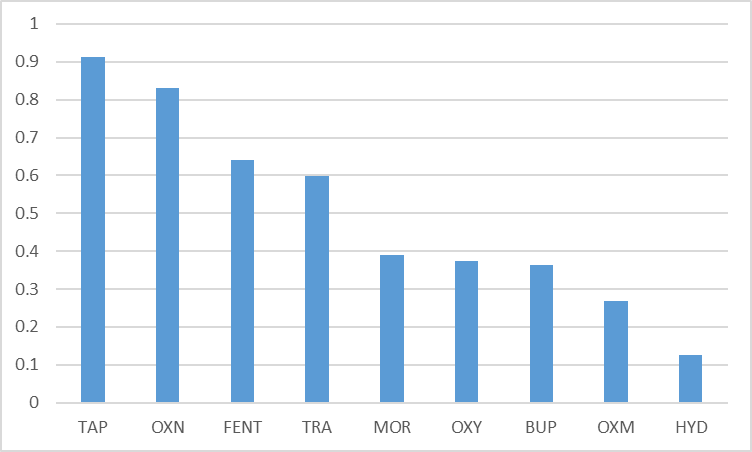 |
| --- | --- |
| A | B |
| 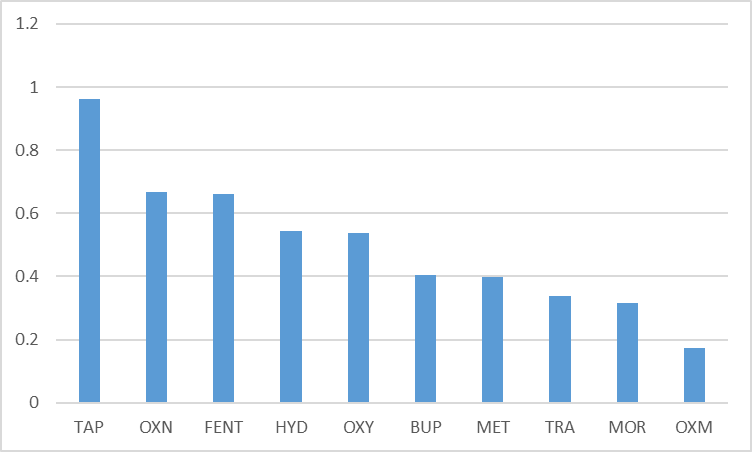 | 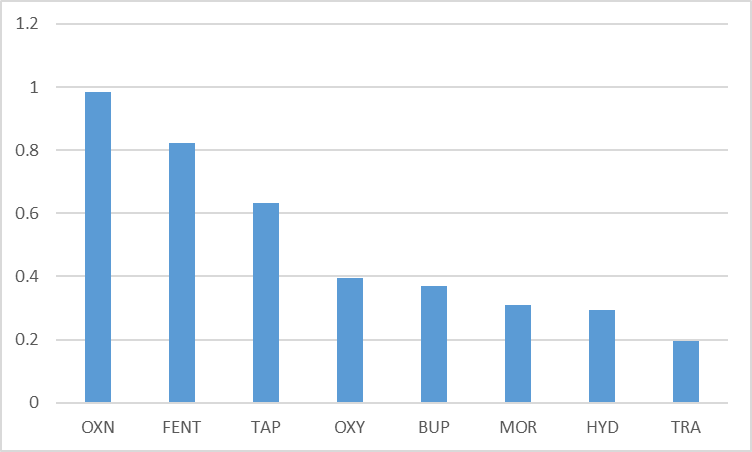 |
| C | D |

Figure S5a-d: Bar charts showing the SUCRA scores of each of the opioid treatment regimen


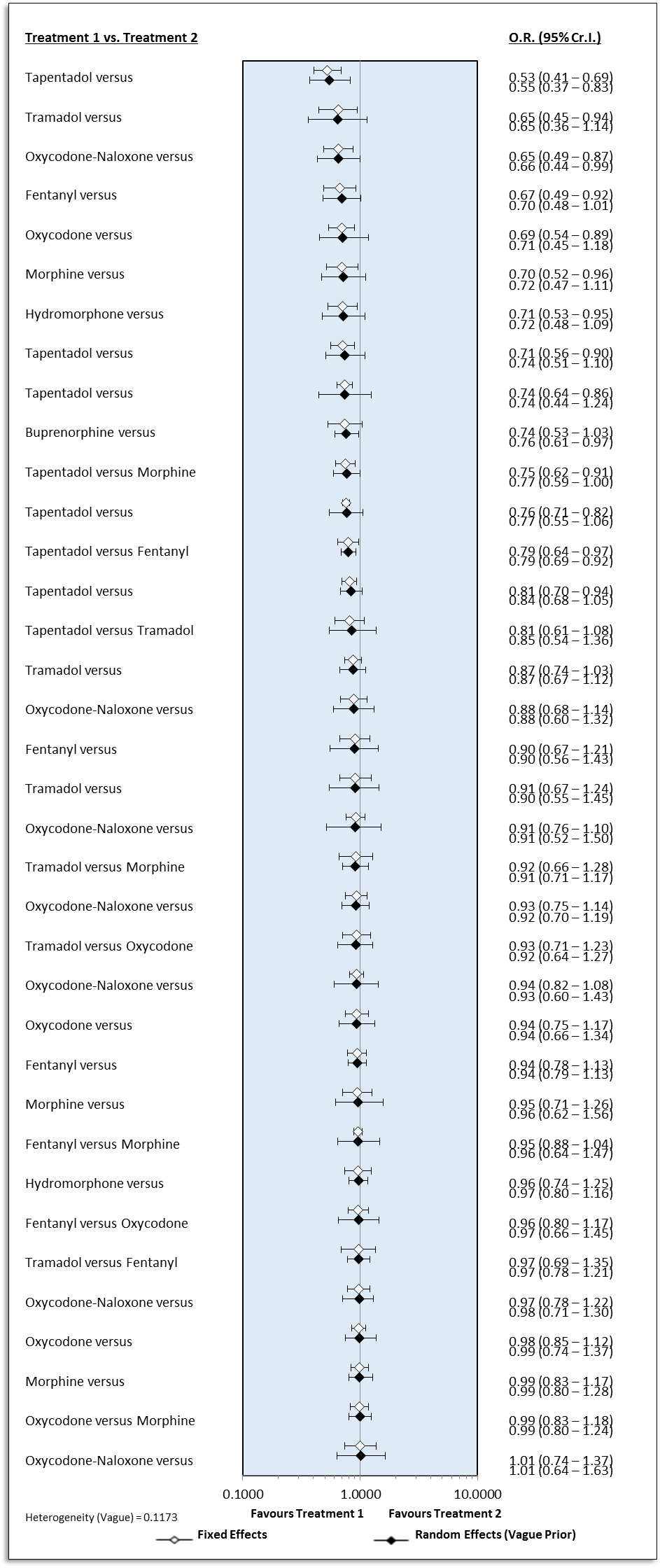


Figure S5a: Forest graph of the network meta-analysis of the odds ratios between various opioids in the incidence of overall **ADVERSE EVENTS.** For each comparison, fixed effects outcomes are given above the random effects outcomes.


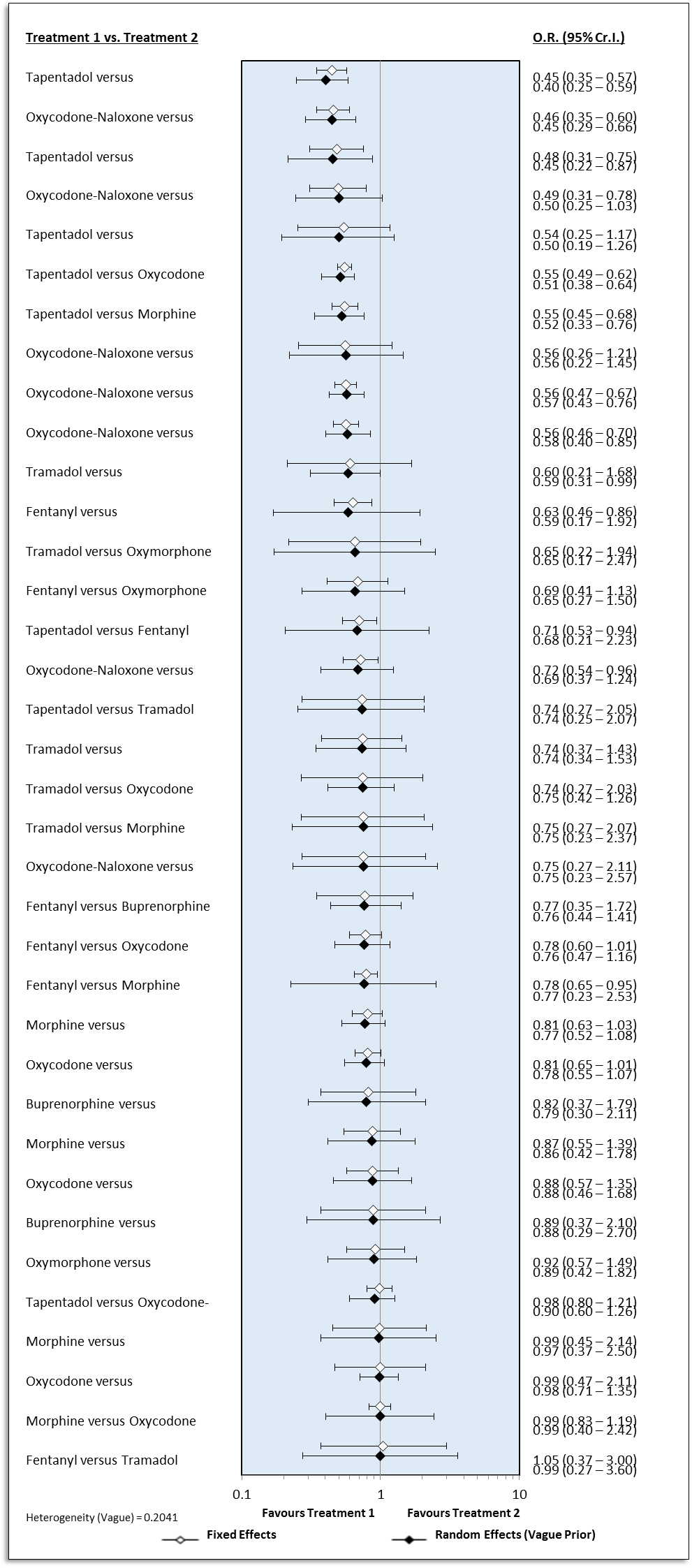


Figure S5b: Forest graph of the network meta-analysis of the odds ratios between various opioids in the incidence of **CONSTIPATION.** For each comparison, fixed effects outcomes are given above the random effects outcomes.


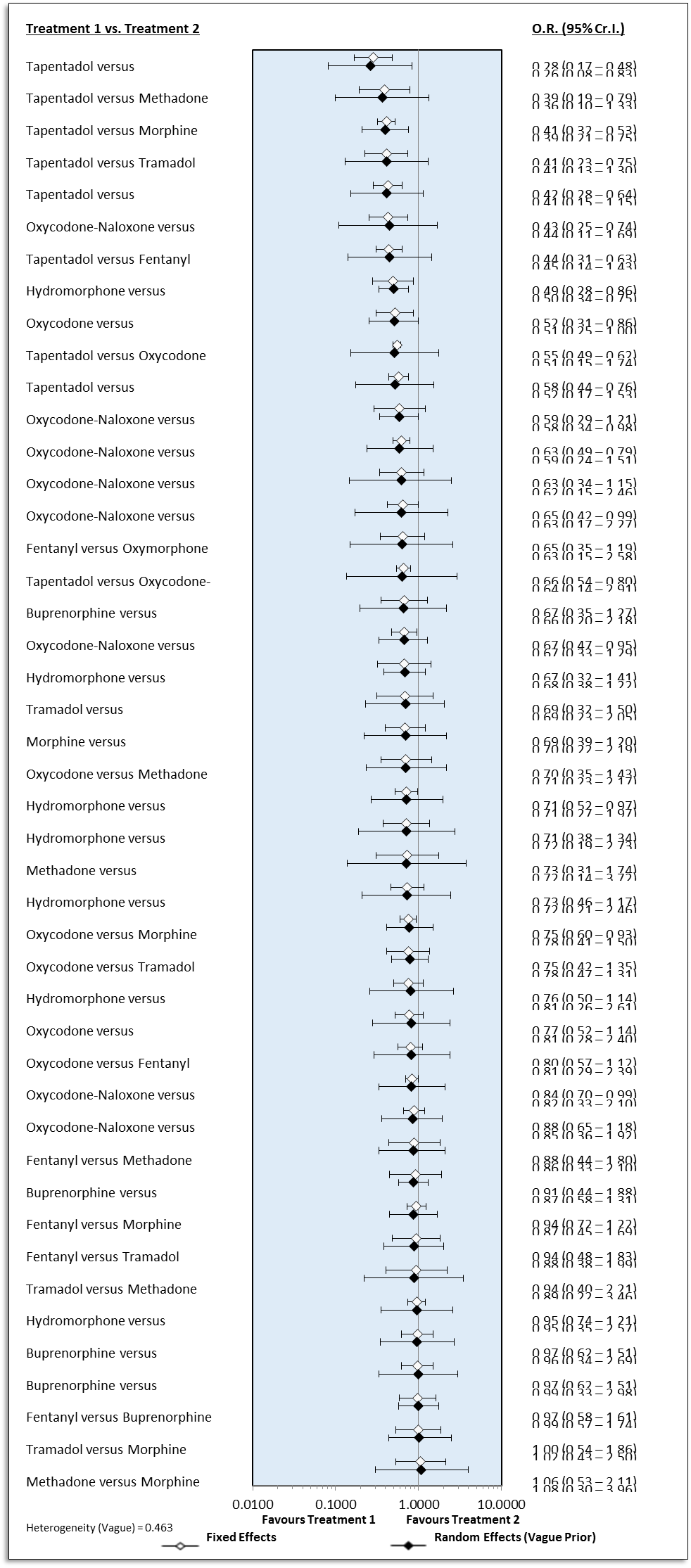


Figure S5c: Forest graph of the network meta-analysis of the odds ratios between various opioids in **TRIAL WITHDRAWAL** rate. For each comparison, fixed effects outcomes are given above the random effects outcomes.


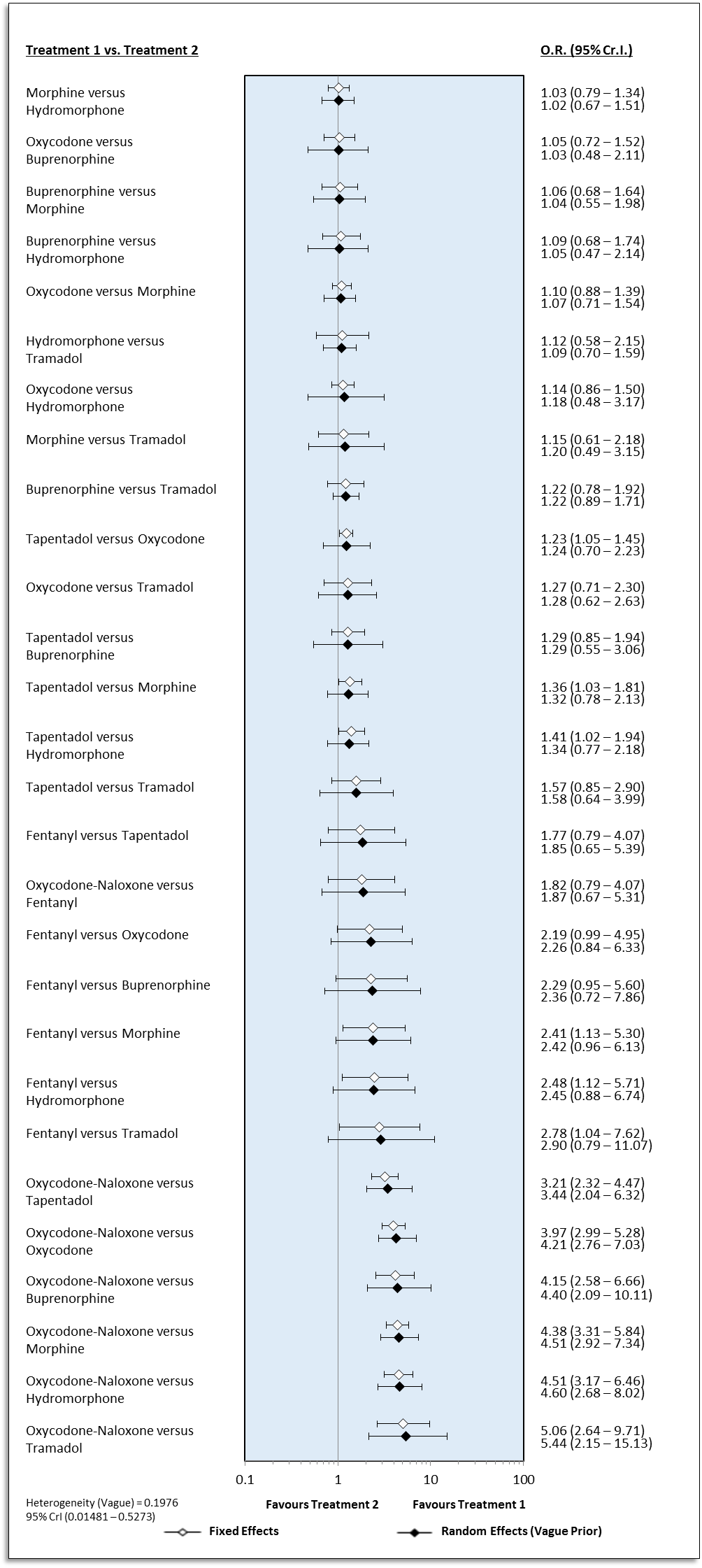


Figure S5d: Forest graph of the network meta-analysis of the odds ratios between various opioids in **PATIENTS’ SATISFACTION.** For each comparison, fixed effects outcomes are given above the random effects outcomes.

**Appendix** 1

| Characteristic of the included studies | | | | | | | | |
| --- | --- | --- | --- | --- | --- | --- | --- | --- |
| Study | **n treated** | **Dose** | **Admn. mode** | **Condition** | **Opioid History** | **Trial duration (weeks)** | **Age** | **% Males** |
| **Ahmedzai 2011** | OXY 92 / OXN 92 | 43.09±19.31 / 46.59±22.58 mg/d | PR vs PR | Cancer | Opioid history | 4 | 63.1±10.2 | 51 |
| **Allan 2001** | MOR 130 / FENT 126 | 133 mg/d / 57.3 µg/h | SR vs TTS | Non-cancer | Opioid history | 4 | 51.5±14 | 53 |
| **Allan 2005** | MOR 342 / FENT 338 | 60 mg/d vs 25 µg/h | SR vs TTS | Low back pain | Naïve | 56 | 54±10.8 | 39 |
| **Baron 2015** | OXN 128 / TAP 130 | 80 mg/d vs 500 mg/d | PR vs PR | Low back pain | Naïve | 9 | 58.2±11.8 | 37 |
| **Binsfeld 2010** | OXY 250 / HYD 254 | 60 mg/d vs 25 µg/h | SR vs ER | LBP/arthritis | Both | 52 | 57.5±12.9 | 42 |
| **Etropolski 2011** | OXY 144 / TAP 154 | 30-40 mg/d vs 300-400 mg/d | IR vs IR | osteoarthritis | Opioid history | 6 | 58.5±8.2 | 44 |
| **Etropolski 2014** | OXY 1237 / TAP 1883 | 40-100mg/d vs 200-500 mg/d | CR vs ER | LBP/osteoarthritis | Both | 12 | 57±12.2 | 38 |
| **Hale 2007** | OXY 69 / HYD 71 | 24±11.7 mg/d vs 15.8±10.5 mg/d | ER vs ER | osteoarthritis | Naïve | 6 | 63.6±12.6 | 30 |
| **Hale 2009** | OXY 170 / TAP 679 | 40-90 mg/d vs 200-600mg/d | IR vs IR | LBP/osteoarthritis | Opioid history | 13 | 56.1±11.9 | 45 |
| **Hanna 2008** | MOR 101 / HYD 99 | 60-540 mg/d vs 12-108 mg/d | CR vs ER | Cancer | Opioid history | 2 | 60±11.9 | 44 |
| **Hanna 2009** | MOR 33 / HYD 35 | 43.7±28.1 mg/d vs NA | CR vs ER | Cancer | Opioid history | 52 | 57.8±12.9 | 37 |
| **Hartick 2009** | OXY 172 / TAP 169 | 40-60 mg/d vs 300-400 mg/d | IR vs IR | Osteoarthritis | Naïve | 10 | 61.8±11 | 52 |
| **Imanaka 2013** | OXY 172 / TAP 171 | 10-80 mg/d vs 50-400 mg/d | CR vs ER | Cancer | Naïve | 4 | 65.2±11.3 | 56 |
| **Karlsson 2009** | BUP 69 / TRA 66 | 10-20 µg/h vs 150-400 mg/d | TTS vs PR | osteoarthritis | Naïve | 12 | 64.3±10.2 | 43 |
| **Kavanagh 2012** | OXY 170 /TAP 678 | 40-90 mg/d vs 200-600 mg/d | IR vs IR | LBP/osteoarthritis | Naive | 13 | 57.7±16 | 45 |
| **Lange 2010** | OXY 1012 / TAP 987 | 40-100 mg/d vs 200-500 mg/d | CR vs PR | LBP/osteoarthritis | Both | 12 | 56.8±12.3 | 37 |
| **Leng 2015** | TRA 139 / BUP 141 | 100-400 mg/d vs 5-20 µg/h | SR vs TTS | Musculoskeletal | Naive | 8 | 57±11 | 31 |
| **Lowenstein 2009** | OXY 295 / OXN 292 | 20-80 mg/d vs 20-120 mg/d | PR vs PR | Non-cancer | Opioid history | 4 | 57.9±11.4 | 36 |
| **Matsumoto 2005** | OXY 125 / OXM 121 | 40 mg/d vs 80mg/d | CR vs ER | osteoarthritis | Opioid history | 4 | 63±1 | 43 |
| **Mercadante 2008** | MOR 22 / FENT 25 / MET 23 | 60 mg/d, 0.6 mg/d, 15 mg/d | SR vs TTS | Cancer | Opioid history | 4 | 59±15 | 54 |
| **Neumann 2013** | BUP-NLX 26 / MET 28 | 16 mg/day–4 mg/d, 20-60 mg/d |  | Non-cancer | Opioid history | 26 | 38.3±9.7 | 54 |
| **Nicholson 2006** | OXY 54 / MOR 43 | 34-85±23-66, 30-79±27-56 mg/d | CR vs ER | Non-cancer | Opioid history | 24 | 51.3±16 | 52 |
| **Richarz 2013** | OXY 52 /HYD 60 | 20-160 mg/d vs 16-64 mg/d | CR vs ER | Non-cancer | Opioid history | 52 | 58.1±11.1 | 46 |
| **Steiner 2011** | OXY 221 / BUP 222 | 40 mg/d vs20 µg/h | IR vs TTS | Low back pain | Opioid history | 12 | 50±12.4 | 52 |
| **Ueberal 2015** | OXY 151 / OXN 144 / MOR 158 | 120±30, 124±25, 127±26 mg total |  | Low back pain | Both | 12 | 46.3±9.7 | 56 |
| **Ueberal 2016** | OXY 300 /OXN 301 / MOR 300 | 107±37, 113±34, 104±39 mg total |  | Low back pain | Both | 12 | 46.4±1 | 56 |
| **van Seventer 2003** | MOR 64 / FENT 67 | 60 mg/d vs 50 µg/h | SR vs TTS | Non-cancer | Naive | 4 | 64.4±11.8 | 65 |
| **Vondrackova 2008** | OXY 151 / OXN 154 | 20-40 mg/d vs 20-40/10-20 mg/d | PR vs PR | Non-cancer | Opioid history | 12 | 56.3±11 | 38 |
| **Wallace 2007** | OXY 60 / HYD 64 | 20-160 mg/d vs 8-64 mg/d | ER vs ER | Non-cancer | Opioid history | 6 | 50±10.8 | 46 |
| **Webster 2006** | OXY 206 / OXN 206 | 10-80 mg/d vs 10-80 mg/d |  | Low back pain | Opioid history | 12 | 47.8±10.8 | 38 |
| **Webster 2013** | OXY 137 / FENT 137 | 15-60 mg/d vs 400-800 µg/d | IR vs Buccal | Cancer/Non-cancer | Opioid history | 12 | 51±10 | 44 |
| **Yu 2014** | OXY 130 / HYD 130 | 38.5±21 mg/d vs 16±8.5 mg/d | CR vs ER | Cancer | Opioid history | 5 | 53±10.7 | 65 |
| **Abbreviations: BUP, buprenorphine; CR, controlled release; ER, extended release; FENT, fentanyl; HYD, hydromorphone; IR, immediate release; MOR, morphine; NLX, naloxone; OXN, oxycodone-naloxone/naltrexone; OXY, oxycodone; PR, prolonged release; SR, sustained release; TAP, tapentadol; TRA, tramadol; TTS, transdermal therapeutic system.** | | | | | | | | |
